# Screening of Benzimidazole-Based Anthelmintics and Their Enantiomers as Repurposed Drug Candidates in Cancer Therapy

**DOI:** 10.3390/ph14040372

**Published:** 2021-04-17

**Authors:** Rosalba Florio, Simone Carradori, Serena Veschi, Davide Brocco, Teresa Di Genni, Roberto Cirilli, Adriano Casulli, Alessandro Cama, Laura De Lellis

**Affiliations:** 1 Department of Pharmacy, G. d’Annunzio University of Chieti-Pescara, 66100 Chieti, Italy; rosalba.florio@unich.it (R.F.); serena.veschi@unich.it (S.V.); davide.brocco@unich.it (D.B.); teresa.digenni@hotmail.it (T.D.G.); laura.delellis@unich.it (L.D.L.); 2 Centro Nazionale per il Controllo e la Valutazione dei Farmaci, Istituto Superiore di Sanità, 00161 Rome, Italy; roberto.cirilli@iss.it; 3 WHO Collaborating Centre for the Epidemiology, Detection and Control of Cystic and Alveolar Echinococcosis (in Animals and Humans), Department of Infectious Diseases, Istituto Superiore di Sanità, 00161 Rome, Italy; adriano.casulli@iss.it; 4 European Union Reference Laboratory for Parasites, Department of Infectious Diseases, Istituto Superiore di Sanità, 00161 Rome, Italy; 5 Center for Advanced Studies and Technology, University “G. d’Annunzio” of Chieti-Pescara, 66100 Chieti, Italy

**Keywords:** benzimidazoles, enantioselective HPLC, pancreatic cancer, paraganglioma, colorectal cancer, drug repositioning, pharmacokinetic parameters, target prediction

## Abstract

Repurposing of approved non-antitumor drugs represents a promising and affordable strategy that may help to increase the repertoire of effective anticancer drugs. Benzimidazole-based anthelmintics are antiparasitic drugs commonly employed both in human and veterinary medicine. Benzimidazole compounds are being considered for drug repurposing due to antitumor activities displayed by some members of the family. In this study, we explored the effects of a large series of benzimidazole-based anthelmintics (and some enantiomerically pure forms of those containing a stereogenic center) on the viability of different tumor cell lines derived from paraganglioma, pancreatic and colorectal cancer. Flubendazole, parbendazole, oxibendazole, mebendazole, albendazole and fenbendazole showed the most consistent antiproliferative effects, displaying IC_50_ values in the low micromolar range, or even in the nanomolar range. In silico evaluation of their physicochemical, pharmacokinetics and medicinal chemistry properties also provided useful information related to the chemical structures and potential of these compounds. Furthermore, in view of the potential repurposing of these drugs in cancer therapy and considering that pharmaceutically active compounds may have different mechanisms of action, we performed an in silico target prediction to assess the polypharmacology of these benzimidazoles, which highlighted previously unknown cancer-relevant molecular targets.

## 1. Introduction

Current anticancer approaches still largely rely on conventional chemotherapy, the efficacy of which is often hampered by the development of drug resistance related to the activation of mechanisms fostering cancer cell survival, metastatic dissemination and immune escape, together with drug efflux and inactivation [1]. Thus, novel and more effective drugs are needed to improve cancer patient outcomes. Unfortunately, the discovery and development of new anticancer drugs, with both improved efficacy in tumors and low toxicity in normal tissues, are tasks of a long, expensive and often challenging process that may fail throughout the clinical trial phases needed before drug approval [2,3]. Intriguingly, the repurposing of non-antitumor drugs to be exploited in cancer therapy represents a valuable and an alternative strategy, since candidate agents have well documented pharmacokinetic and pharmacodynamic features, together with good safety profiles, which may speed up their approval and implementation in the clinics [4,5]. Moreover, the development and marketing of inexpensive, already-approved products to be used for a different therapeutic indication is a relevant strategy for an orphan designation, or for reducing costs that healthcare systems have to sustain for warranting access of patients to therapies. In this regard, benzimidazole-based anthelmintics are among the non-anticancer drugs that have been considered as candidates for repurposing in oncology because of pre-clinical evidence supporting their antitumor properties in several cell and animal models [6,7,8,9]. Specifically, several studies showed that some members of the benzimidazole-based anthelmintic family, such as albendazole, mebendazole and flubendazole, have a remarkable antitumor activity in melanoma, leukemia, medulloblastoma, breast and colorectal cancers [10,11,12,13,14,15,16]. In addition, we recently reported that parbendazole, mebendazole, fenbendazole and oxibendazole also display antitumor effects in pancreatic cancer cells [17]. It is worth noting that, similarly to other repurposed drugs [18,19], several pathways that are crucial in cancer biology have been reported to be modulated by benzimidazoles, which explains their antitumor activities [20,21]. In this regard, compounds that affect multiple targets might have improved effectiveness and ability to overcome acquired resistance to conventional chemotherapy.

**Figure 1:**
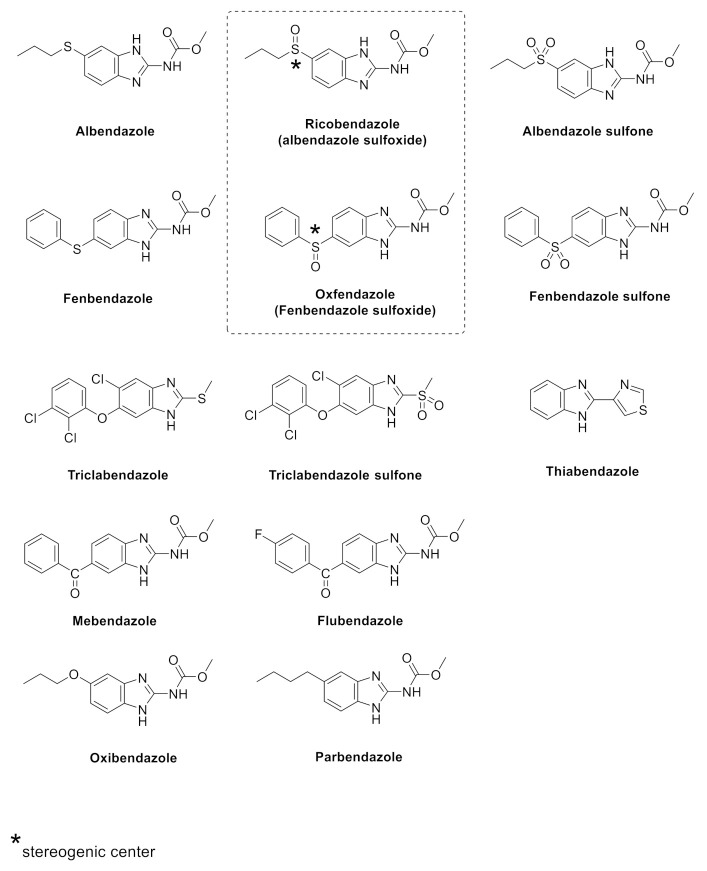
Structure of the screened benzimidazoles. For chiral compounds, the single enantiomers, (*R*)-ricobendazole/(*S*)-ricobendazole and (*R*)-oxfendazole/(*S*)-oxfendazole, respectively, were obtained and tested.

Based on these considerations, in the present study we explored the antiproliferative effects of a wider series of benzimidazole-based anthelmintics against a panel of cell lines representative of three different tumors, including pancreatic (AsPC-1 and BxPC-3), paraganglioma (PTJ64i and PTJ86i) and colorectal (HT-29 and SW480) cancer cell lines, in order to expand the available arsenal of chemotherapeutic agents. Notably, the two unique cell lines PTJ64i and PTJ86i were previously established in our laboratory from two patients with paraganglioma [22,23], a rare tumor that is poorly responsive to standard chemotherapy, for which new therapeutic agents are urgently needed, and which may benefit from an orphan designation. Unfortunately, this task remains largely unexplored in paraganglioma, also due to the lack of commercial cell lines. It is worth noting that we included in the series of tested molecules two couples of enantiomers derived from the corresponding two chiral benzimidazoles (i.e., ricobendazole and oxfendazole) to analyze whether they could have distinct effects on the viability of the aforementioned cancer cell lines based on the stereoselective metabolism involving this organic scaffold (Figure 1). In the series, we also tested the antiproliferative activities against parasites of new benzimidazole salt formulations, recently licensed by some of us, which demonstrated improved bioavailability and solubility in vivo, in studies against secondary cystic echinococcosis using experimentally infected mice [24]. Remarkably, these patented salts were prepared to overcome the issues related to the poor aqueous solubility of the benzimidazole scaffold and the corresponding erratic bioavailability, as previously explored also by other authors through several strategies, including the inclusion in cyclodextrin complexes [25,26], the transformation into soluble prodrugs [27], the characterization and use of selected polymorphic forms and solvates [28], and the preparation of innovative formulations [29]. In addition to testing antiproliferative activities, we also predicted physico-chemical properties and putative molecular targets for our series of benzimidazoles, using an in silico approach, to further explore the polypharmacology of these compounds.

## 2. Results and Discussion

**Figure 2:**
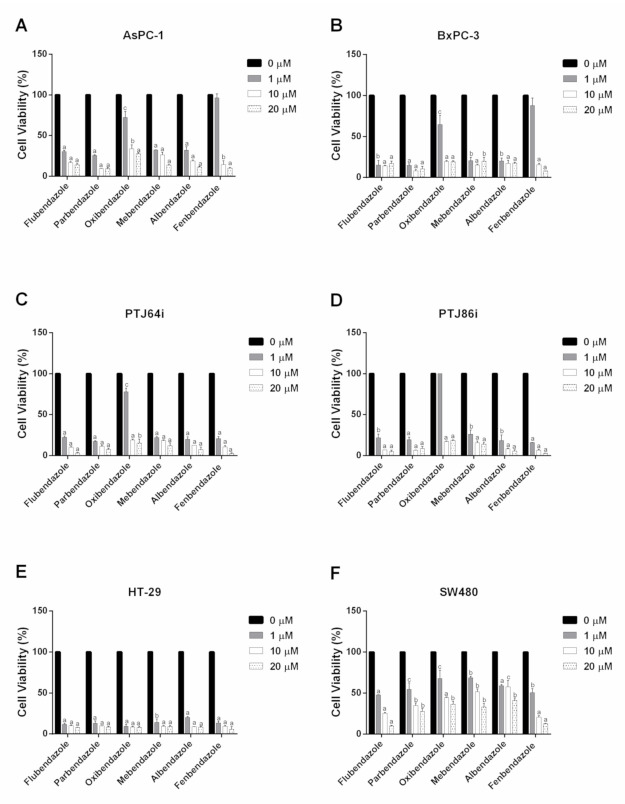
Effects of flubendazole, parbendazole, oxibendazole, mebendazole, albendazole and fenbendazole on the viability of pancreatic (**A**,**B**), paraganglioma (**C**,**D**) and colorectal (**E**,**F**) cancer cell lines. Cell viability was evaluated by 72 h MTT assays using different concentrations of the drugs, as indicated. Data shown are the means ± SD of two to four independent experiments with quintuplicate determinations. Statistically significant differences between control (0 μM) and each drug concentration are reported (c = *p* < 0.05; b = *p* < 0.01; a = *p* < 0.001).

The effects of 15 benzimidazole-based anthelmintics (Figure 1) on the viability of six cancer cell lines (AsPC-1, BxPC-3, PTJ64i, PTJ86i, HT-29 and SW480) were analyzed by MTT. The experiments were conducted by incubating cell lines for 72 h with benzimidazoles at concentrations ranging from 0 μM (vehicle) to 20 μM. Figure 2 and Table 1 show the results obtained with the compounds most consistently active across the tested cancer cell lines, namely flubendazole, parbendazole, oxibendazole, mebendazole, albendazole, and fenbendazole.

Specifically, the six most potent compounds had half maximal inhibitory concentration (IC_50_) values ranging from 0.01 μM to 3.26 μM, with inhibition rates from 72% to 92% in the two pancreatic cancer cell lines (Figure 2A,B and Table 1). In line with these results, the same benzimidazoles displayed IC_50_ values ranging from 0.01 μM to 3.29 μM and inhibition rates from 82% to 97%, also in paraganglioma cell lines (Figure 2C,D and Table 1). Notably, in the latter cells, flubendazole and fenbendazole showed the most drastic and consistent effects on cell viability at 20 μM, with inhibition rates ranging from 95% to 97% (Figure 2C,D). In terms of colorectal cancer cell lines, the six compounds showed the lowest IC_50_ values, which ranged from 0.01 μM to 1.26 μM, but they exhibited variable potency in the two cell lines (Figure 2E,F and Table 1). In fact, inhibition of cell viability was more marked in HT-29, where most compounds had the lowest IC_50_ values, together with high inhibition rates ranging from 91% to 94% (Figure 2E and Table 1). Conversely, in SW480, the six benzimidazoles showed less consistent effects. In particular, parbendazole, oxibendazole, mebendazole and albendazole induced a less pronounced reduction in cell viability at 20 μM (inhibition rates from 59% to 72%), whereas flubendazole and fenbendazole affected cell viability with inhibition rates of 91% and 87%, respectively (Figure 2F and Table 1). In this regard, it should be noted that low IC_50_ values do not always reflect the maximum inhibition of cell viability, since a potent effect achieved at a lower concentration might not be always followed by a greater effect at higher concentrations of a drug.

Interestingly, flubendazole, parbendazole, oxibendazole, mebendazole, albendazole and fenbendazole affected viability across the cancer cell lines with IC_50_ values within the range of, or even lower than, the plasma concentrations achieved by the drugs at standard therapeutic doses, thus supporting their relevance in the context of clinical translation [7,30].

With respect to ricobendazole and oxfendazole, key metabolites of albendazole and fenbendazole, respectively, they present a stereogenic center (e.g., the sulfur atom of sulfonyl moiety) and, consequently, consist of an equimolar mixture of enantiomers. As for other chiral pharmaceutical molecules, the enantiomers of two sulfoxides may display different pharmacological properties. In this regard, in vivo studies showed that the antiparasitic activity of the (*R*)-ricobendazole enantiomer is higher than the (*S*)-ricobendazole counterpart [24]. Moreover, an accumulation of (*R*)-ricobendazole has been observed in the cerebrospinal fluid of patients with neurocysticercosis [31]. This stereoselectivity in the drug–organism interaction prompted us to explore the in vitro antiproliferative activity of sulfoxides using their individual enantiomers rather than racemic forms.

**Figure 3:**
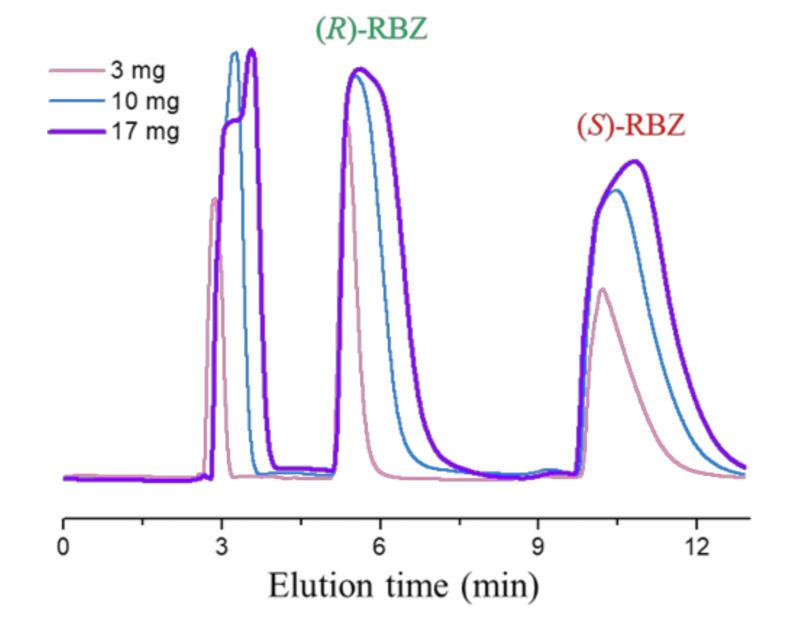
Loading study of ricobendazole (**RBZ**) on the Chiralpak IG column. Chromatographic conditions: column, Chiralpak IG (250 mm × 10 mm i.d.); mobile phase, methanol-acetonitrile 50:50; flow rate, 5.5 mL/min; column temperature, 40 °C; detection: UV at 310 nm.

We have previously described the HPLC separation of the enantiomers of the chiral sulfoxides ricobendazole and oxfendazole on polysaccharide-based chiral stationary phases [32]. In particular, good enantioseparations could be achieved using the immobilized amylose-based Chiralpak IG chiral stationary phase in normal phase and polar organic conditions [32]. In the present study, a 250 mm × 10 mm i.d. Chiralpak IG column [33,34] was employed, in order to optimize and isolate multi-milligram quantities of enantiopure forms of ricobendazole (as also previously reported for oxfendazole). The chromatograms depicted in Figure 3 are representative of increasing amounts of ricobendazole (RBZ) resolved in a single chromatographic run. Notably, for a sample loading of 17 mg of ricobendazole dissolved in 5 mL of methanol/acetone 10:1 (*v*/*v*), the baseline fractionation of two enantiomers was possible.

The two enantiomers were isolated with enantiomeric excess (ee) >99% and in 90–95% recovery. Thus, considering that the analysis time was 12 min, a total of 240 mg of racemic sample could be resolved in 24 h. It is worth highlighting that the first collected enantiomer has (*R*) absolute configuration, which is the stereochemistry described to implement the activity of potential new drugs for human helminthiases.

**Figure 4:**
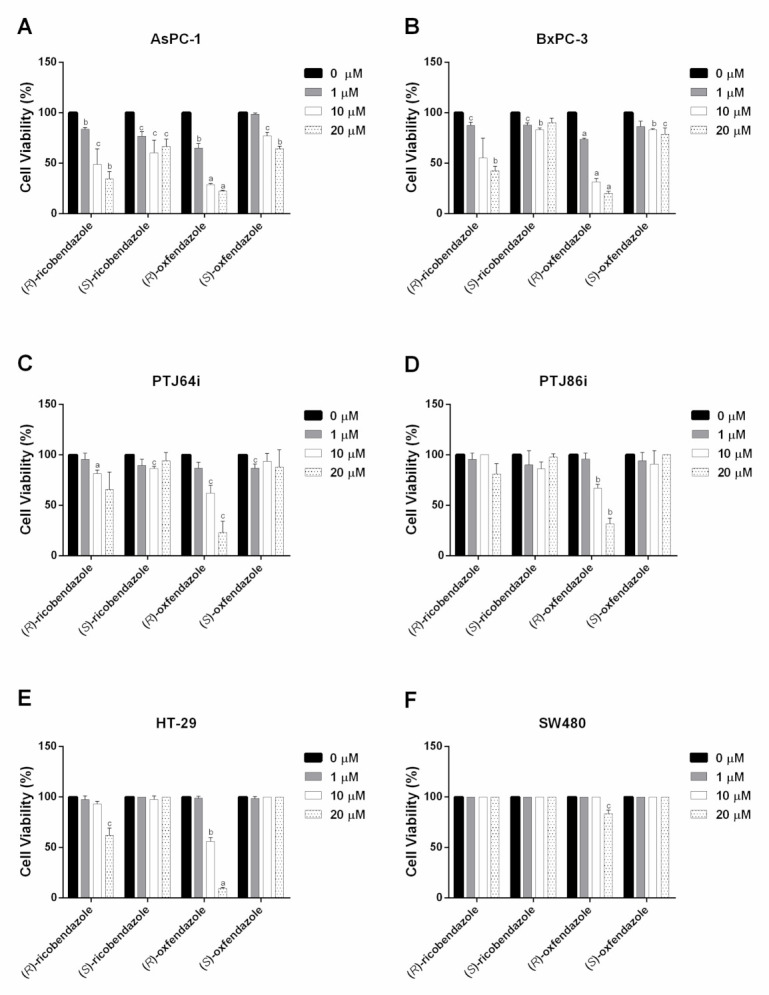
Effect of individual enantiomers (*R*)-ricobendazole/(*S*)-ricobendazole and (*R*)-oxfendazole/(*S*)-oxfendazole on the viability of pancreatic (**A**,**B**), paraganglioma (**C**,**D**) and colorectal (**E**,**F**) tumor cell lines. Cell viability was evaluated by 72 h MTT assays using different concentrations of the drugs, as indicated. Data shown are the means ± SD of two to three independent experiments with quintuplicate determinations. Statistically significant differences between control (0 μM) and each drug concentration are reported (c = *p* <0.05; b = *p* < 0.01; a = *p* < 0.001).

Overall, the two pairs of enantiomers exhibited less prominent and homogeneous antiproliferative effects against the six cancer cell lines, as compared to the most active analogues in Table 1 (Figure 4 and Table 2). Of note, (*R*)-ricobendazole and (*R*)-oxfendazole induced a pronounced inhibition of viability in AsPC-1 and BxPC-3 pancreatic cancer cells, with IC_50_ values ranging from 1.18 μM to 13.6 μM (Table 2 and Figure 4A,B). In addition, (*R*)-oxfendazole was also consistently active in reducing the viability of PTJ64i e PTJ86i paraganglioma cell lines, with IC_50_ values of 10.02 μM and 12.41 μM, respectively, and in HT-29 colorectal cancer cell line, displaying an IC_50_ of 10.02 μM. On the other hand, none of the enantiomers had IC_50_ values lower than 20 μM in SW480 colorectal cancer cell line, suggesting that this cell line is quite resistant to this series of compounds (Figure 4 and Table 2). Overall, it should be noted that both (*S*)-ricobendazole and (*S*)-oxfendazole had poor inhibitory activity on the viability of the six tested cell lines, as indicated by their IC_50_ values all being greater than 20 μM, indicating that the antiproliferative effects of the two (*R*)-enantiomers are consistently more marked than those of their (*S*)-enantiomer counterparts in the panel of cancer cell lines.

Lastly, thiabendazole, triclabendazole and the sulfones of triclabendazole, albendazole and fenbendazole showed very poor inhibitory effects on cancer cell viability, displaying IC_50_ values that were all higher than 20 μM, which was the highest concentration used in the MTT assays (Table S1).

The use of saline forms of each compound, with improved aqueous solubility which enables them to be more easily administered as oral doses in further in vivo and clinical studies, is among the main strengths of this study. It should be noted that the compounds presented in the patent were obtained by means of a direct and cheap modification of the parent compounds and they display better handling in the performance of in vitro studies and HPLC analyses. Moreover, for the first time, the chiral compounds were separated and tested against distinct cancer cell lines, in order to gain information about the antiproliferative activities of these metabolites. These data have an impact on the knowledge of the pharmacokinetic data of the parent drugs. Remarkably, benzimidazoles had never been tested before in paraganglioma cell lines and several of them drastically and significantly decreased paraganglioma cell line viability, which is a relevant result in the search for effective compounds in the treatment of this rare tumor, which is poorly responsive to standard chemotherapy.

### In Silico Pharmacokinetic Parameters and Target Prediction

To gain further insights into the underexplored potential of benzimidazole-based anthelmintics as candidates for repurposing in cancer therapy, we analyzed these compounds using the web tool SwissADME. This bioinformatic tool allowed us to assess important information regarding both the chemical structure and polypharmacology of these agents, according to the classic Medicinal Chemistry principles. In this regard, we were able to compute some parameters describing the physical–chemical, pharmacokinetic and medicinal chemistry properties of the most active compounds flubendazole, parbendazole, oxibendazole, mebendazole, albendazole and fenbendazole (Table 3). The drug-likeness of the compounds was evaluated keeping in mind the Lipinski’s rule of five, which was met by all the derivatives that also exhibited a high likelihood of being passively adsorbed in the gastrointestinal tract, thus supporting their use as oral drugs in humans. An interesting prevision proposed by the SwissADME tool refers to the absence of interaction with permeability glycoprotein (P-gp) that promotes the efflux of cytotoxic drugs out of the cells, which in turn reduces the efficacy of antitumor treatments. P-gp is overexpressed in tumor cells and is involved in multi-drug resistance [35]. Interestingly, none of the evaluated compounds seemed to be substrates of that protein (Table 3).

In addition, the presence of substructures able to elicit promiscuous pharmacological behaviour, commonly named PAINS (Pan Assay Interference Compounds), was also evaluated (Table 3). This analysis showed that all compounds are devoid of this property. The same data were also obtained for all the remaining compounds and are reported in Table S2.

**Figure 5:**
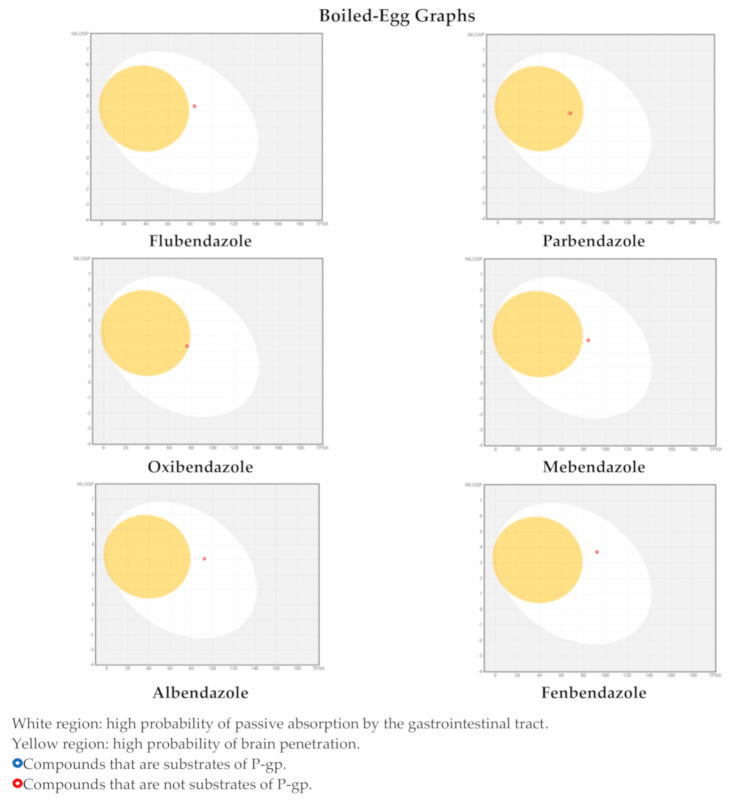
Boiled-egg graphs calculated by SwissADME web-tool for the compounds flubendazole, parbendazole, oxibendazole, mebendazole, albendazole, and fenbendazole.

The boiled-egg images, one of the two graphical outputs of the SwissADME tool along with the bioavailability-radar, are reported in Figure 5 for the six aforementioned benzimidazoles. These graphs were obtained by considering the parameters WLOGP and TPSA reported in Table 4, which are a lipophilic index and a measure of apparent polarity, respectively. These graphs allow an easy understanding and visualization of two ADME parameters that are the passive absorption at the GI tract (white area) and the ability to permeate the blood–brain barrier (BBB, yellow area). Furthermore, for thoroughness of information, the susceptibility to P-gp was also reported, depending on the colour of the dot (red dot: the compound is not a substrate of the P-gp; blue dot: the compound is a substrate of the P-gp). As one can see, only two compounds, namely parbendazole and oxibendazole, were estimated to be able to cross the BBB, as would be required to reach central nervous system tumors, whereas all the others exhibited passive absorption at the GI level. The dot colour indicates that all the compounds are not (theoretically) substrates of P-gp, as previously assessed in Table 3.

**Figure 6:**
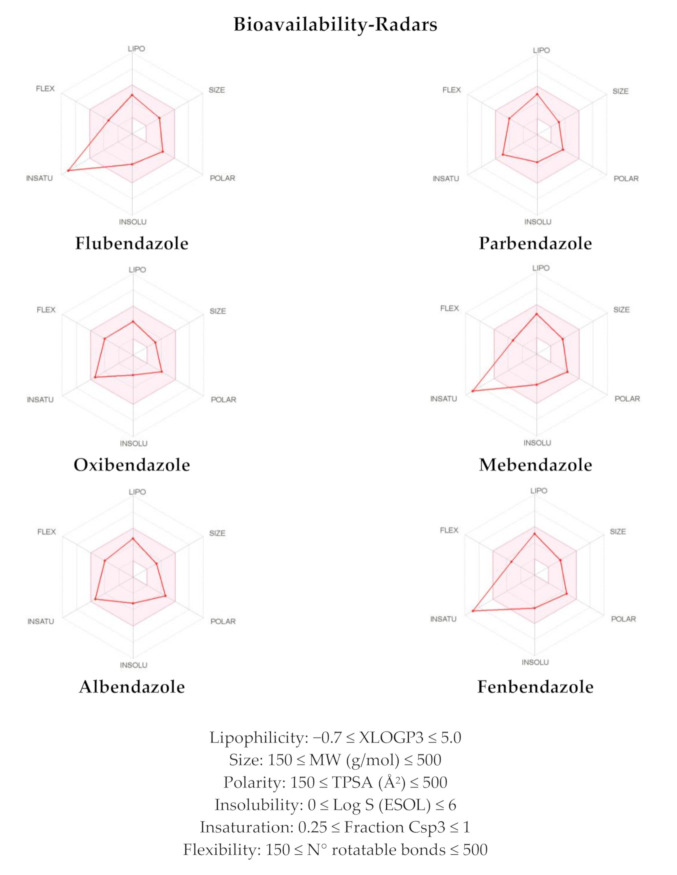
Bioavailability-radars computed by the SwissADME web-tool for the compounds flubendazole, parbendazole, oxibendazole, mebendazole, albendazole, and fenbendazole.

The bioavailability radars reported in Figure 6 offer the drug-likeness depiction of the selected compounds. In the graphs, the pink-coloured area includes the optimal range of each physical–chemical property (lipophilicity, size, polarity, solubility, saturation and flexibility) that is essential for oral bioavailability and, therefore, potentially active in tumors outside the gastrointestinal tract. Parbendazole, oxibendazole and albendazole exhibited all these properties inside the desired range, thus accounting for the good oral biovailability. Conversely, flubendazole, mebendazole, and fenbendazole exhibited the insaturation parameter out of the red-depicted area, suggesting a putative moderate oral bioavailability (Figure 6). The results of these analyses for the remaining benzimidazole derivatives have been reported in Table S3 and Figures S1 and S2.

We also exploited the web-tool SwissTargetPrediction to unravel alternative target/s for our compounds (Table 5 and Table 6 and Table S4). We performed the analysis on all the compounds, paying particular attention to the most promising targets predicted for each one. Notably, for the two derivatives fenbendazole and mebendazole, we obtained interesting results in terms of high probability scores for a few cancer-related targets (Table 5 and Table 6).

Fenbendazole was predicted to be able to interact with MAP kinase p38 alpha, whose dysregulation was associated with advanced stages and short survival in cancer patients (Table 5) [36]. On the other hand, for mebendazole, presumed interactions with tyrosine-protein kinase ABL and vascular endothelial growth factor receptor 2 were recognized as the most probable ones (Table 6). Tyrosine-protein kinase ABL plays a crucial role in different key processes related to cell growth and survival in response to extracellular stimuli. Its overexpression is often found in chronic myeloid leukemia (CML) [37]. Vascular endothelial growth factors (VEGFs) control the vascular development through the binding with different VEGF receptors (VEGFR). Among them, the VEGFR-2 regulates vascular endothelial functions through its intracellular signaling cascades, which induces proliferation, migration, survival and increased permeability, thus promoting angiogenic response [38]. Angiogenesis is a crucial step for tumor development because cancer cells have high metabolic needs of oxygen and nutrients required to feed the abnormal growth. So, the inhibition of the VEGF-2 receptor could be effective in impairing tumor vascularization, also promoting a “normal” vasculature within the tumor and leading to the efficient delivery of antitumor drugs. 

Surprisingly, although the mammalian tubulin is present within the SwissADME protein database, it was not pinpointed by the web tool as a target for benzimidazoles, despite several studies reporting its relevance as a molecular target for explaining the antitumor properties of these compounds [12,16,17]. However, it must be considered that repurposed drugs may modulate multiple pathways in cancer cells; thus, it is interesting that additional cancer-relevant targets, distinct from tubulin, emerged in SwissADME target prediction, which may contribute to the anticancer actions of benzimidazoles. Thus, it will be mandatory to validate the predicted, previously unknown, molecular targets of these compounds in future in vitro and in vivo studies, in view of potential, valuable polypharmacological profiles of benzimidazoles to be exploited in cancer treatment.

## 3. Materials and Methods

### 3.1. Cell Cultures and Treatments

Human pancreatic cancer cell lines AsPC-1 and BxPC-3, human colorectal cancer cell lines SW480 and HT-29, human PTJ64i and PTJ86i paraganglioma cell lines were cultured as previously described [39,40,41]. Benzimidazole-based anthelmintics were purchased from Sigma-Aldrich (Milan, Italy) and salified as licensed [42]. All drugs were dissolved in DMSO and then diluted to the final working concentrations in culture media.

### 3.2. Cell Viability Assay

Cell viability was assessed by MTT assay (Sigma-Aldrich, St. Louis, MO, USA), as previously described [43]. Specifically, MTT assays were performed by exposing cancer cell lines to benzimidazoles at the indicated concentrations (1, 10, 20 μM) or with vehicle DMSO (0 μM).

### 3.3. IC_50_ Calculation and Statistical Analysis

IC_50_ values were calculated by GraphPad 7 (GraphPad Software, San Diego, CA, USA). Statistical analyses were performed by comparing mean values using an unpaired Student’s t-test. A *p*-value ≤ 0.05 was estimated as statistically significant.

### 3.4. Enantioselective HPLC

Ricobendazole, oxfendazole and HPLC-grade solvents were purchased from Sigma-Aldrich (Milan, Italy) and used without further purification. HPLC enantioseparations were performed by using stainless-steel Chiralpak IG (250 mm × 10 mm I.D.) columns (Chiral Technologies Europe, Illkirch, France). The HPLC apparatus for semipreparative separations consisted of a Perkin-Elmer (Norwalk, CT, USA) 200 LC pump equipped with a Rheodyne (Cotati, CA, USA) injector, a 5000 μL sample loop, a Perkin-Elmer LC 101 oven and a Waters 484 detector (Waters Corporation, Milford, MA, USA). Data were processed using Clarity software (DataApex, Prague, Czech Republic).

### 3.5. In silico Analysis

In silico analysis of the most active compounds was performed using SwissADME, a free online software application that allows the evaluation of the pharmacokinetics, as well as the drug-likeness (the probability of being an oral drug) and medicinal chemistry friendliness (PAINS) of small molecules [44]. Target prediction was attempted, taking advantage of the SwissTargetPrediction web-tool [45].

## 4. Conclusions

Several benzimidazole-based anthelmintic compounds have been recognized to exert antitumor effects with a marked cancer cell-specific selectivity. In the present study, we investigated the effects of a large series of benzimidazole-based anthelmintics and their enantiomers on the viability of different tumor cells, including paraganglioma, and pancreatic and colorectal cancer cell lines. Six benzimidazoles, namely flubendazole, parbendazole, oxibendazole, mebendazole, albendazole and fenbendazole, emerged as the most active compounds, with consistent antiproliferative effects across the tested cancer cell lines and IC_50_ values all in the low micromolar range, or even in the nanomolar range. Interestingly, in silico evaluation of their physicochemical, pharmacokinetics and medicinal chemistry properties predicted the potential of these six compounds as candidates for repurposing as oral drugs for cancer in humans. Moreover, these analyses predicted the absence of interaction of the six most potent benzimidazoles with P-gp permeability glycoprotein, responsible for drug efflux in tumors, and a moderate to good oral bioavailability. Notably, for the two derivatives fenbendazole and mebendazole, target prediction analysis pointed out a few cancer-related molecular targets having very high probability scores, thus suggesting polypharmacological profiles of these drugs. These bioinformatic predictions will have to be validated in future studies to expand our insights into the therapeutic relevance of benzimidazole-based anthelmintics as candidates for repurposing in cancer therapy.

## Figures and Tables

**Table 1:** IC_50_ values of the six most active benzimidazoles on pancreatic, paraganglioma and colorectal cancer cell lines.

Compound	IC_50_ (μM)					
	Pancreatic Cancer		Paraganglioma		Colorectal Cancer	
	AsPC-1	BxPC-3	PTJ64i	PTJ86i	HT-29	SW480
**Flubendazole**	0.23	0.01	0.14	0.25	0.01	0.67
**Parbendazole**	0.58	0.03	0.04	0.17	0.01	0.57
**Oxibendazole**	1.45	1.07	1.79	3.29	0.01	0.96
**Mebendazole**	0.08	0.40	0.01	0.19	0.08	1.26
**Albendazole**	0.19	0.10	0.05	0.29	0.28	0.17
**Fenbendazole**	3.26	2.66	0.10	0.15	0.02	0.78

**Table 2:** IC_50_ values of individual enantiomers (*R*)-ricobendazole/(*S*)-ricobendazole and (*R*)-oxfendazole/(*S*)-oxfendazole on pancreatic, paraganglioma and colorectal cancer cell lines.

Compound	IC_50_ (μM)					
	Pancreatic Cancer		Paraganglioma		Colorectal Cancer	
	AsPC-1	BxPC-3	PTJ64i	PTJ86i	HT-29	SW480
***(R*)-ricobendazole**	9.09	13.6	>20	>20	>20	>20
**(*S*)-ricobendazole**	>20	>20	>20	>20	>20	>20
***(R*)-oxfendazole**	1.18	1.82	10.02	12.41	10.02	>20
**(*S*)-oxfendazole**	>20	>20	>20	>20	>20	>20

**Table 3:** In silico evaluated physicochemical properties of flubendazole (**FLU**), parbendazole (**PAR**), oxibendazole (**OXI**), mebendazole (**MEB**), albendazole (**ALB**) and fenbendazole (**FEN**).

Physicochemical Properties	FLU	PAR	OXI	MEB	ALB	FEN
**Molecular Weight (MW)**	313.28	247.29	249.27	295.29	265.33	299.35
**H-Bond Acceptors (HBA)**	5	3	4	4	3	3
**H-Bond Donators (HBD)**	2	2	2	2	2	2
Consensus Log P *	2.56	2.52	1.85	2.26	2.29	2.91
**Lipinski Violations**	0	0	0	0	0	0
**GI Absorption**	High	High	High	High	High	High
**P-gp Substrate**	No	No	No	No	No	No
**PAINS Alerts**	0	0	0	0	0	0

* Arithmetic means of the values predicted by five in silico methods: XLOGP3, WLOGP, MLOGP, SILICOS-IT and iLOGP. Parameters range required to satisfy the Lipinski’s rule of five: MW < 500 g/mol, HBD < 5, HBA < 10, log *P* < 5.

**Table 4:** In silico estimated physicochemical parameters of the compounds flubendazole (**FLU**), parbendazole (**PAR**), oxibendazole (**OXI**), mebendazole (**MEB**), albendazole (**ALB**) and fenbendazole (**FEN**).

Cmpd	WLOGP ^a^	TPSA (Å^2^) ^a,b^	XLOGP3 ^b^	Log S (ESOL) ^b^	MW ^b^	Csp3 ^b^	No. of Rotatable Bonds ^b^
**FLU**	3.34	84.08	2.84	−3.72	313.28	0.06	5
**PAR**	2.89	67.01	3.28	−3.41	247.29	0.38	6
**OXI**	2.34	76.24	2.27	−2.79	249.27	0.33	6
**MEB**	2.78	84.08	3.01	−3.74	295.29	0.06	5
**ALB**	3.05	92.31	2.81	−3.23	265.33	0.33	6
**FEN**	3.70	92.31	3.47	−4.08	299.35	0.07	5

^a^ Parameters used to generate the boiled-egg graphs (Figure 5). ^b^ Parameters used to generate the bioavailability radars (Figure 6). Bioavailability radar parameters’ functional ranges: XLOGP3, between −0.7 and +5.0; MW, between 150 and 500 g/mol; TPSA, between 20 and 130 Å^2^, log S not higher than 6; saturation: fraction of carbons in the sp^3^ hybridization not less than 0.25; flexibility: no more than 9 rotatable bonds.

**Table 5:** The best results obtained by protein target prediction for fenbendazole.

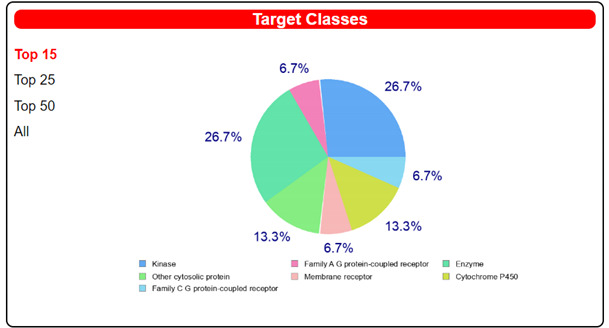			
Target	Common Name	Target Class	Probability
MAP kinase p38 alpha	MAPK14	Kinase	1
Adenosine A2a receptor	ADORA2A	Family A G protein-coupled receptor	0.13079
1-acylglycerol-3-phosphate *O*-acyltransferase beta	AGPAT2	Enzyme	0.09787

**Table 6:** The best results obtained by protein target prediction for mebendazole.

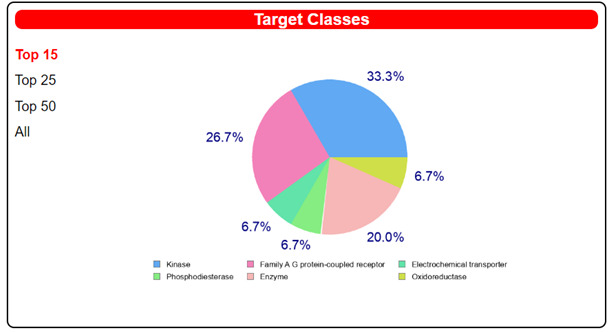			
Target	Common Name	Target Class	Probability
Tyrosine-protein kinase ABL	ABL1	Kinase	1
Vascular endothelial growth factor receptor 2	KDR	Kinase	1
Adenosine A2a receptor	ADORA2A	Family A G protein-coupled receptor	0.1194

## Data Availability

Data are contained within the article.

## Supplementary Materials

The following are available online at https://www.mdpi.com/article/10.3390/ph14040372/s1. Table S1. IC_50_ values of the six less active benzimidazoles on pancreatic (AsPC-1, BxPC-3), paraganglioma (PTJ64i, PTJ86i), and colorectal (HT-29, SW480) cancer cell lines. These IC_50_ values were calculated by Graphpad PRISM software after a 72 h treatment with benzimidazoles at concentrations ranging from 0 μM to 20 μM and were all higher than 20 μM, which was the highest concentration used in MTT assays. Table S2. Physicochemical properties of albendazole sulfone (ALB.SO_2_), fenbendazole sulfone (FEN.SO_2_), thiabendazole (THI), triclabendazole (TRI), triclabendazole sulfone (TRI.SO_2_), ricobendazole (RBZ), and oxfendazole (OXF), evaluated using an in-silico approach. Table S3. In silico estimated physicochemical parameters of compounds albendazole sulfone (ALB.SO_2_), fenbendazole sulfone (FEN.SO_2_), thiabendazole (THI), triclabendazole (TRI), triclabendazole sulfone (TRI.SO_2_), ricobendazole (RBZ), and oxfendazole (OXF), used to device the boiled-egg graph (Figure S1) and bioavailability radar (Figure S2). Table S4. Results obtained by protein target prediction for (1) albendazole, (2) ricobendazole, (3) albendazole sulfone, (4) oxfendazole, (5) fenbendazole sulfone, (6) triclabendazole, (7) triclabendazole sulfone, (8) thiabendazole, (9) flubendazole, (10) oxibendazole, and (11) parbendazole. Figure S1. Representation of the boiled-egg graph calculated using the SwissADME web-tool, for the compounds albendazole sulfone, fenbendazole sulfone, thiabendazole, triclabendazole, triclabendazole sulfone, ricobendazole, and oxfendazole. Figure S2. Representation of the bioavailability-radar computed using the SwissADME web-tool, for the compounds albendazole sulfone, fenbendazole sulfone, thiabendazole, triclabendazole, triclabendazole sulfone, ricobendazole, and oxfendazole.
