# Supplementary material for: Screening of Benzimidazole-Based Anthelmintics and Their Enantiomers as Repurposed Drug Candidates in Cancer Therapy
Source: Pharmaceuticals (Basel). 2021 Apr 17;14(4):372. doi: 10.3390/ph14040372 (PMC8072969; doi:10.3390/ph14040372)
Supplement: Supplementary file 1 [file pharmaceuticals-14-00372-s001.zip › pharmaceuticals-1175891-SI.pdf]

Supplementary Information

Screening of benzimidazole-based anthelmintics and their enantiomers as repurposed drug candidates in cancer therapy

Rosalba Florio[1], Simone Carradori[1,*], Serena Veschi[1], Davide Brocco[1], Teresa Di Genni[1], Roberto Cirilli[2], Adriano Casulli[3,4], Alessandro Cama[1,5,*], Laura De Lellis[1]

[1]Department of Pharmacy, G. d'Annunzio University of Chieti-Pescara, 66100 Chieti, Italy

[2]Centro nazionale per il controllo e la valutazione dei farmaci, Istituto Superiore di Sanità, 00161 Rome, Italy

[3]WHO Collaborating Centre for the Epidemiology, Detection and Control of Cystic and Alveolar Echinococcosis (in Animals and Humans), Istituto Superiore di Sanità (ISS), 00161 Rome, Italy

[4]European Union Reference Laboratory for Parasites, Istituto Superiore di Sanità (ISS), 00161 Rome, Italy

[5]Center for Advanced Studies and Technology (CAST), University "G. d'Annunzio" of Chieti-Pescara, 66100 Chieti, Italy

*Corresponding authors: Simone Carradori PhD, simone.carradori@unich.it; Alessandro Cama, MD, alessandro.cama@unich.it

Table S1. IC_{50} values of the six less active benzimidazoles on pancreatic (AsPC-1, BxPC-3), paraganglioma (PTJ64i, PTJ86i), and colorectal (HT-29, SW480) cancer cell lines. These IC_{50} values were calculated by Graphpad PRISM software after a 72-hour treatment with benzimidazoles at concentrations ranging from 0 µM to 20 µM and resulted all higher than 20 µM, which was the highest concentration used in MTT assays.

	**IC_{50} (µM)**					
	**AsPC-1**	**BxPC-3**	**PTJ64i**	**PTJ86i**	**HT-29**	**SW480**
**Thiabendazole**	>20	>20	>20	>20	>20	>20
**Triclabendazole**	>20	>20	>20	>20	>20	>20
**Triclabendazole sulfone**	>20	>20	>20	>20	>20	>20
**Albendazole sulfone**	>20	>20	>20	>20	>20	>20
**Fenbendazole sulfone**	>20	>20	>20	>20	>20	>20

Table S2. Physicochemical properties of albendazole sulfone (**ALB.SO$_2$**), fenbendazole sulfone (**FEN.SO$_2$**), thiabendazole (**THI**), triclabendazole (**TRI**), triclabendazole sulfone (**TRI.SO$_2$**), ricobendazole (**RBZ**), and oxfendazole (**OXF**) evaluated using an *in silico* approach.

Compound	ALB.SO$_2$	FEN.SO$_2$	THI	TRI	TRI.SO$_2$	RBZ	OXF
**H-bond acceptors (HBA)**	5	5	2	2	4	4	4
**H-bond donators (HBD)**	2	2	1	1	1	2	2
**Consensus Log P***	1.52	2.04	2.33	4.88	4.04	1.59	2.12
**Lipinski violations**	0	0	0	1	0	0	0
**GI absorption**	High	High	High	High	High	High	High
**P-gp substrate**	No	No	Yes	No	No	No	No
**PAINS alerts**	0	0	0	0	0	0	0

*Arithmetic mean of the values predicted by five *in silico* methods: XLOGP3, WLOGP, MLOGP, SILICOS-IT, iLOGP. Parameters range required to satisfy the Lipinski's rule of five: MW $\leq$ 500 g/mol, HBD $\leq$ 5, HBA $\leq$ 10, log P $\leq$ 5.

Table S3. *In silico* estimated physicochemical parameters of compounds albendazole sulfone (**ALB.SO$_2$**), fenbendazole sulfone (**FEN.SO$_2$**), thiabendazole (**THI**), triclabendazole (**TRI**), triclabendazole sulfone (**TRI.SO$_2$**), ricobendazole **(RBZ**), and oxfendazole (**OXF**), used to device the boiled-egg graph (Figure S1) and bioavailability radar (Figure S2).

Cmpd	WLOGP[a]	TPSA (Å^2)[a,b]	XLOGP3[b]	Log S (ESOL)[b]	MW[b]	Csp3[b]	N° of rotatable bonds[b]
**ALB.SO$_2$**	2.81	109.53	1.53	-2.58	297.33	0.33	6
**FEN.SO$_2$**	3.46	109.53	2.20	-3.43	331.35	0.07	5
**THI**	2.69	69.81	2.22	-3.16	201.25	0	1
**TRI**	6.04	63.21	5.74	-6.02	359.66	0.07	3
**TRI.SO$_2$**	5.80	80.43	4.47	-5.37	391.66	0.07	3
**RBZ**	2.93	103.29	1.45	-2.45	281.33	0.33	6
**OXF**	3.58	103.29	2.18	-3.34	315.35	0.07	5

[a]Parameters used for the boiled-egg graph. [b]Parameters used for the bioavailability radar. Bioavailability radar parameters functional ranges: XLOGP3 between −0.7 and +5.0, MW between 150 and 500 g/mol, TPSA between 20 and 130 Å^2, log S not higher than 6, saturation: fraction of carbons in the sp^3 hybridization not less than 0.25, and flexibility: no more than 9 rotatable bonds.

Table S4. Results obtained by protein target prediction for (1) albendazole, (2) ricobendazole, (3) albendazole sulfone, (4) oxfendazole, (5) fenbendazole sulfone, (6) triclabendazole, (7) triclabendazole sulfone, (8) thiabendazole, (9) flubendazole, (10) oxibendazole, and (11) parbendazole.

1. Albendazole

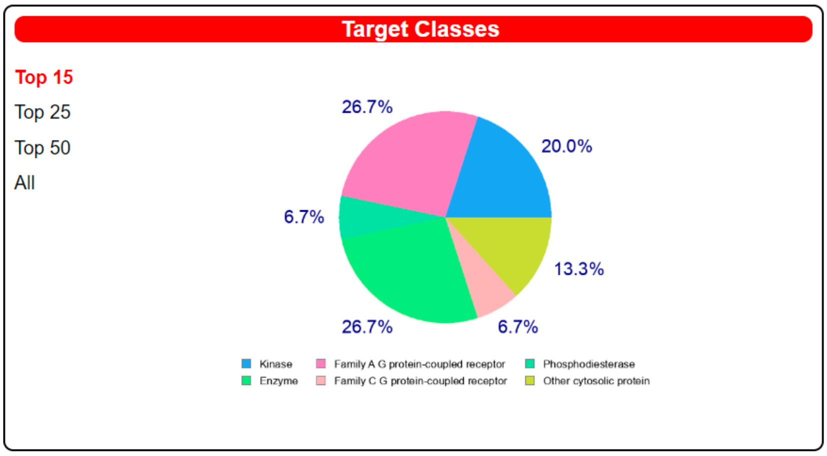

Target	Common name	Target Class	Probability
MAP kinase p38 alpha	MAPK14	Kinase	0.14433
Adenosine A2a receptor	ADORA2A	Family A G protein-coupled receptor	0.06262
Serine/threonine-protein kinase Aurora-A	AURKA	Kinase	0.05356
Adrenergic receptor beta	ADRB2	Family A G protein-coupled receptor	0.05356
Beta-1 adrenergic receptor	ADRB1	Family A G protein-coupled receptor	0.05356

2. Ricobendazole

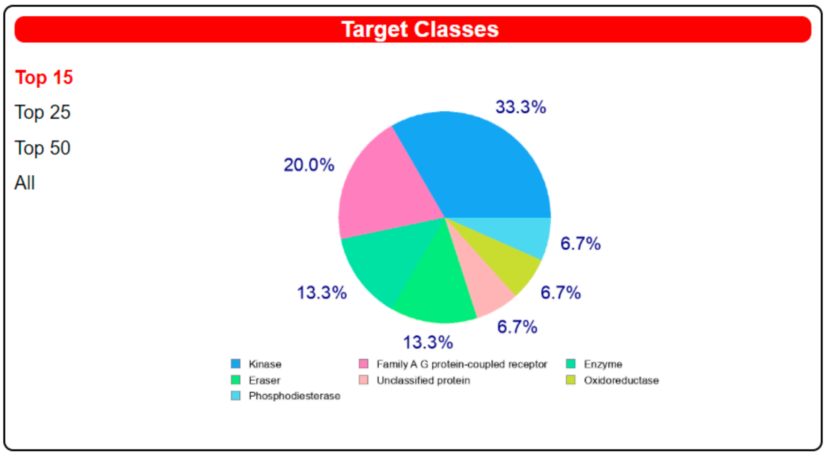

Target	Common name	Target Class	Probability
MAP kinase p38 alpha	MAPK14	Kinase	0.12775
Adenosine A2a receptor	ADORA2A	Family A G protein-coupled receptor	0.12775
Poly [ADP-ribose] polymerase-1	PARP1	Enzyme	0.11204
Histone deacetylase 6	HDAC6	Eraser	0.11204
Histone deacetylase 1	HDAC1	Eraser	0.11204

3. Albendazole sulfone

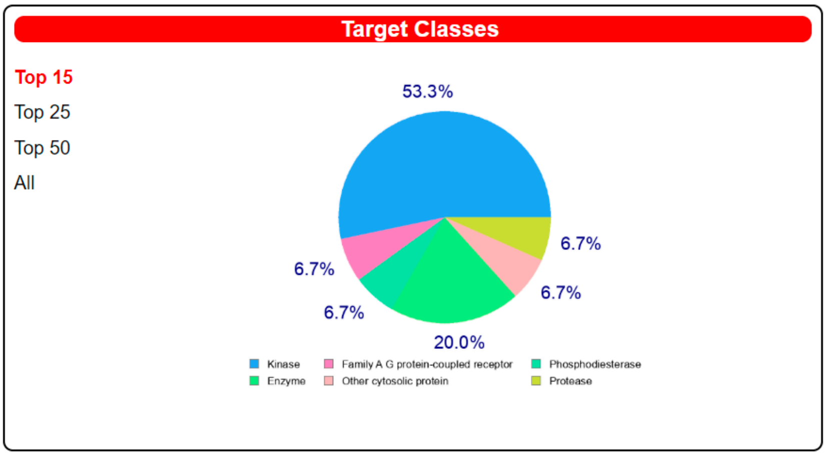

Target	Common name	Target Class	Probability
MAP kinase p38 alpha	MAPK14	Kinase	0.10058
Adenosine A2a receptor	ADORA2A	Family A G protein-coupled receptor	0.10058
Tyrosine-protein kinase SRC	SRC	Kinase	0.10058
Phosphodiesterase 4A	PDE4A	Phosphodiesterase	0.10058
Cyclin-dependent kinase 5/CDK5 activator 1	CDK5R1 CDK5	Kinase	0.10058

4. Oxfendazole

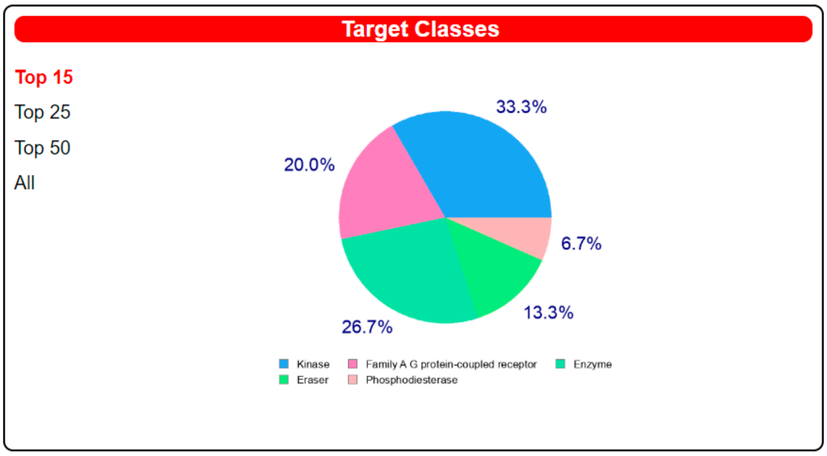

Target	Common name	Target Class	Probability
Adenosine A2a receptor	ADORA2A	Family A G protein-coupled receptor	0.41168
MAP kinase p38 alpha	MAPK14	Kinase	0.25369
11-beta-hydroxysteroid dehydrogenase 1	HSD11B1	Enzyme	0.1115
Poly [ADP-ribose] polymerase-1	PARP1	Enzyme	0.1115
Cannabinoid receptor 1	CNR1	Family A G	0.1115

		protein-coupled receptor	

5. Fenbendazole sulfone

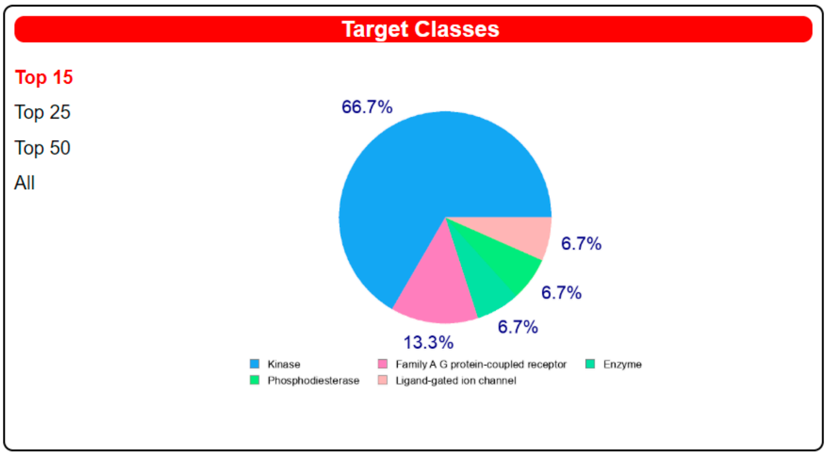

Target	Common name	Target Class	Probability
MAP kinase p38 alpha	MAPK14	Kinase	0.21825
Adenosine A2a receptor	ADORA2A	Family A G protein-coupled receptor	0.10995
1-acylglycerol-3-phosphate *O*-acyltransferase beta	AGPAT2	Enzyme	0.10161
Receptor protein-tyrosine kinase erbB-2	ERBB2	Kinase	0.10161
Epidermal growth factor receptor erbB1	EGFR	Kinase	0.10161

6. Triclabendazole

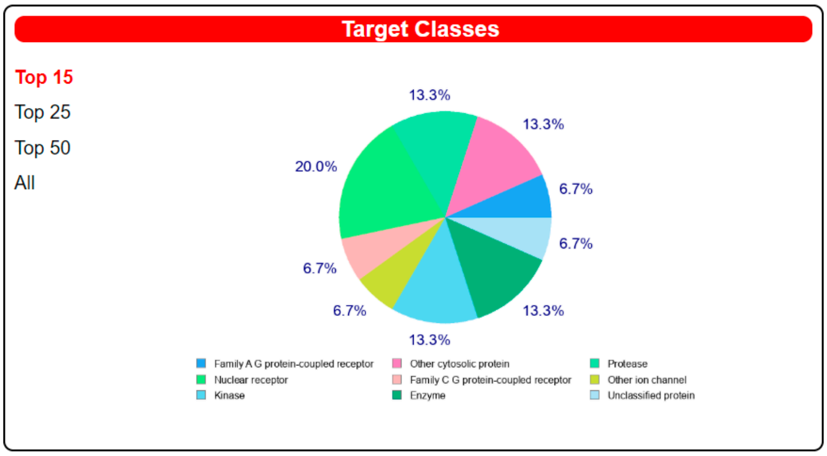

Target	Common name	Target Class	Probability
Adenosine A1 receptor	ADORA1	Family A G protein-coupled receptor	0.09787
Heat shock protein HSP 90-beta	HSP90AB1	Other cytosolic	0.09787

		protein	
Cathepsin K	CTSK	Protease	0.09787
Cathepsin S	CTSS	Protease	0.09787
Mineralocorticoid receptor	NR3C2	Nuclear receptor	0.09787

7. Triclabendazole sulfone

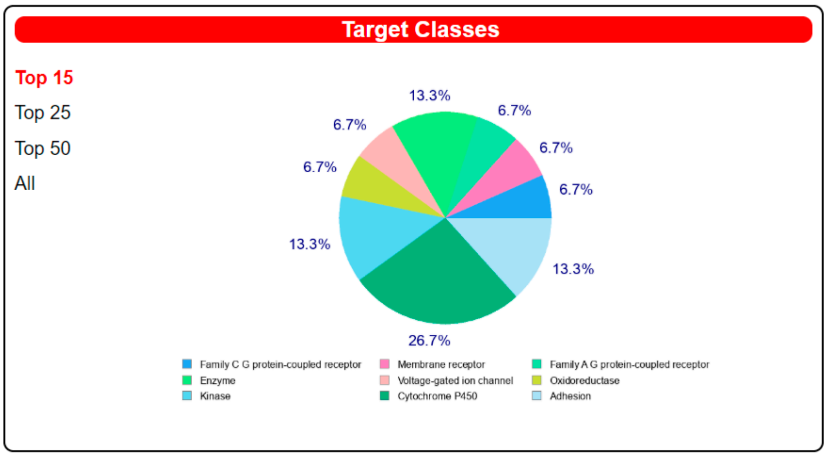

Target	Common name	Target Class	Probability
Metabotropic glutamate receptor 5	GRM5	Family C G protein-coupled receptor	0.10161
Tumor necrosis factor receptor R1	TNFRSF1A	Membrane receptor	0.10161
Adenosine A3 receptor	ADORA3	Family A G protein-coupled receptor	0.10161
11-beta-hydroxysteroid dehydrogenase 1	HSD11B1	Enzyme	0.10161
Transient receptor potential cation channel subfamily A member 1	TRPA1	Voltage-gated ion channel	0.10161

8. **Thiabendazole**

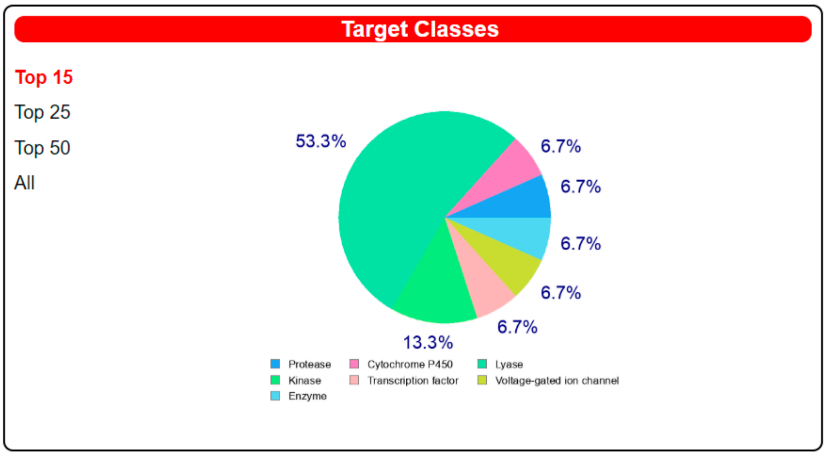

Target	Common name	Target Class	Probability
Methionine aminopeptidase 1	METAP1	Protease	0.9888
Cytochrome P450 1A2	CYP1A2	Cytochrome P450	0.9888
Carbonic anhydrase II	CA2	Lyase	0.03123
Carbonic anhydrase I	CA1	Lyase	0.03123
Carbonic anhydrase IX	CA9	Lyase	0.03123

9. **Flubendazole**

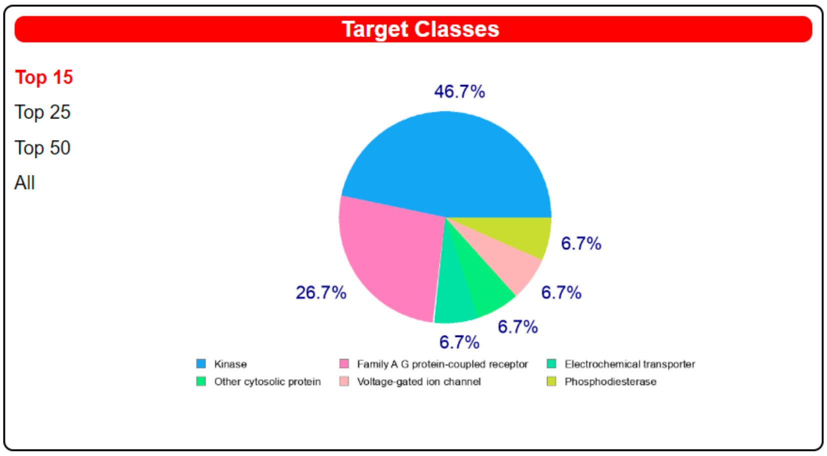

Target	Common name	Target Class	Probability
Tyrosine-protein kinase ABL	ABL1	Kinase	0.50156
Vascular endothelial growth factor receptor 2	KDR	Kinase	0.50156
Adenosine A2a receptor	ADORA2A	Family A G protein-coupled receptor	0.10161
Dopamine transporter	SLC6A3	Electrochemical transporter	0.10161
Glycogen synthase kinase-3 beta	GSK3B	Kinase	0.10161

10. Oxibendazole

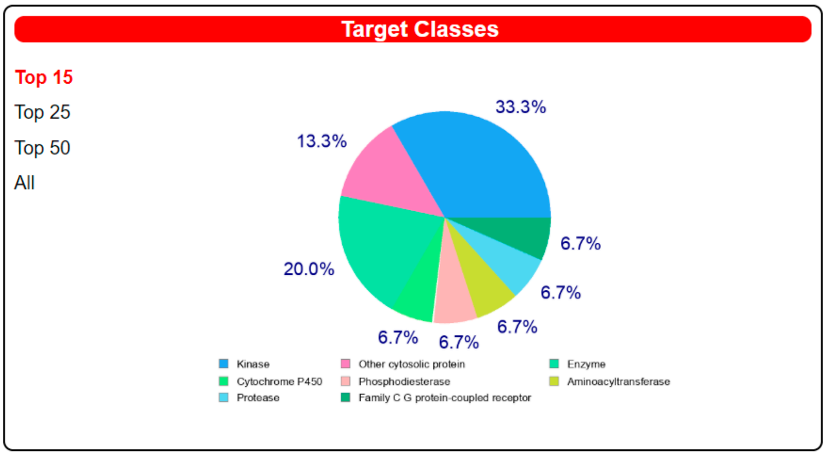

Target	Common name	Target Class	Probability
Vascular endothelial growth factor receptor 2	KDR	Kinase	0.05356
Tyrosine-protein kinase JAK1	JAK1	Kinase	0.05356
Tyrosine-protein kinase JAK2	JAK2	Kinase	0.05356
Tyrosine-protein kinase TYK2	TYK2	Kinase	0.05356
Tyrosine-protein kinase TIE-2	TEK	Kinase	0.05356

11. Parbendazole

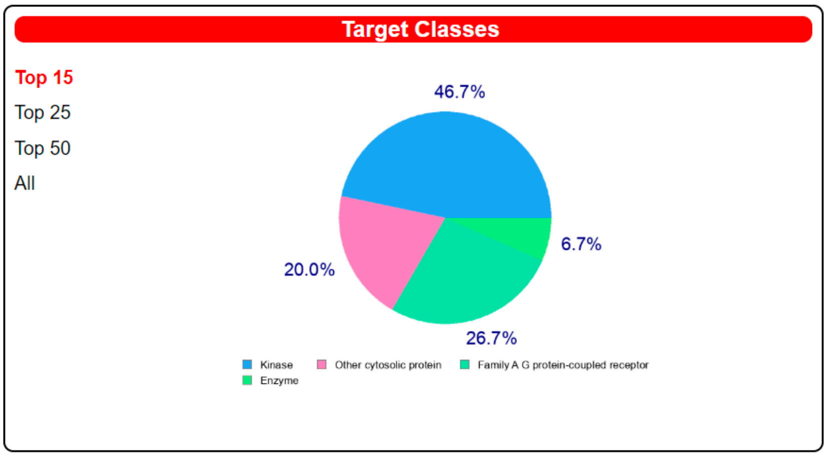

Target	Common name	Target Class	Probability
Tyrosine-protein kinase ABL	ABL1	Kinase	0.08079
Vascular endothelial growth factor receptor 2	KDR	Kinase	0.06262
Serine/threonine-protein kinase Aurora-A	AURKA	Kinase	0.05356
5-lipoxygenase activating protein	ALOX5AP	Other cytosolic protein	0.05356
Tyrosine-protein kinase SRC	SRC	Kinase	0.05356

Figure S1. Representation of the boiled-egg graph calculated by SwissADME web-tool, for the compounds albendazole sulfone, fenbendazole sulfone, thiabendazole, triclabendazole, triclabendazole sulfone, ricobendazole, and oxfendazole.

Boiled-egg graphs

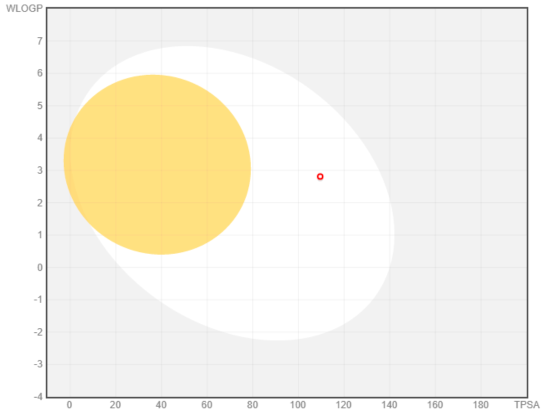

Albendazole sulfone

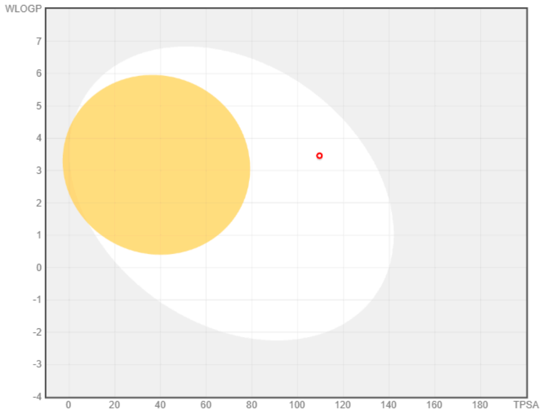

Fenbendazole sulfone

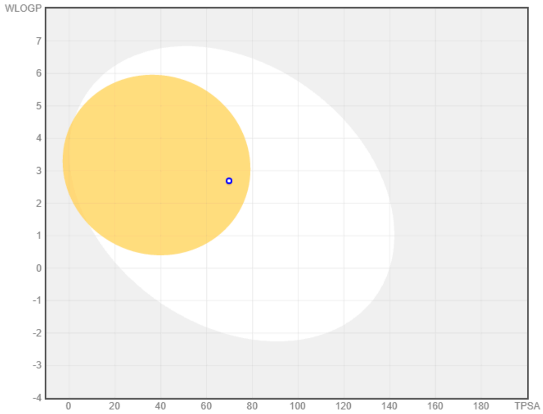

Thiabendazole

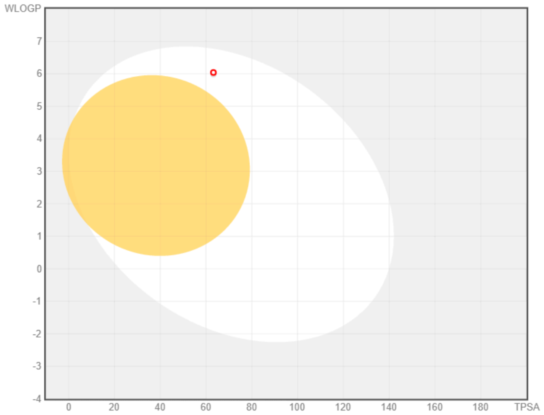

Triclabendazole

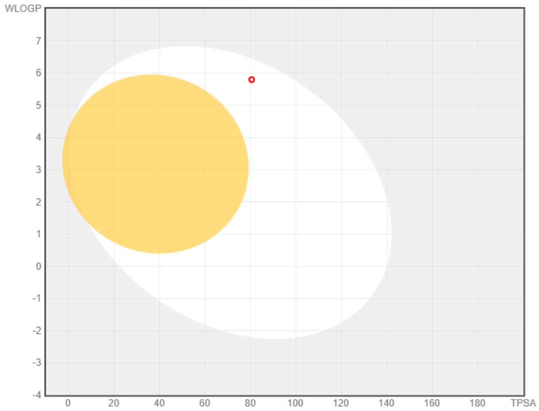

Triclabendazole sulfone

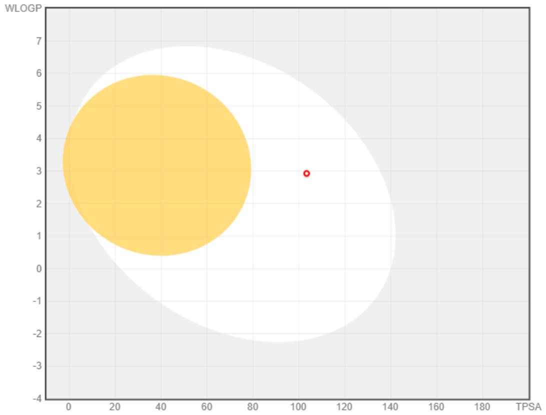

Ricobendazole

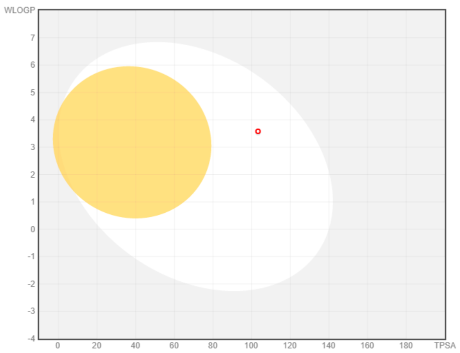<br>**Oxfendazole**	**White region**: high probability of passive absorption by the gastrointestinal tract.<br>**Yellow region**: high probability of brain penetration.<br>Compounds that are substrates of P-gp<br>Compounds that are not substrates of P-gp

Figure S2. Representation of the bioavailability-radar computed by SwissADME web-tool, for the compounds albendazole sulfone, fenbendazole sulfone, thiabendazole, triclabendazole, triclabendazole sulfone, ricobendazole, and oxfendazole.

<table>
<tr><th colspan="2">Bioavailability-radar</th></tr>
<tr>
<td>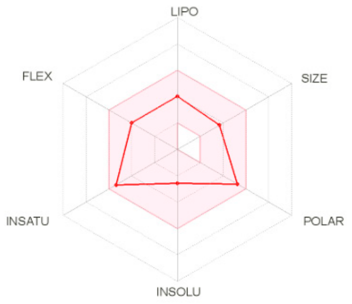

Albendazole sulfone</td>
<td>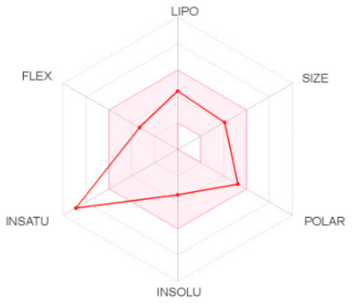

Fenbendazole sulfone</td>
</tr>
<tr>
<td>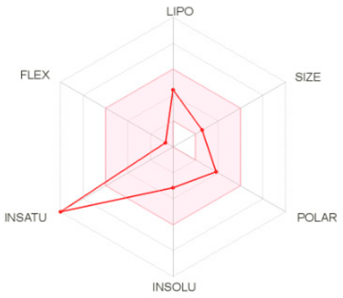

Thiabendazole</td>
<td>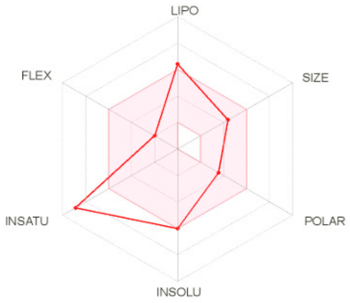

Triclabendazole</td>
</tr>
<tr>
<td>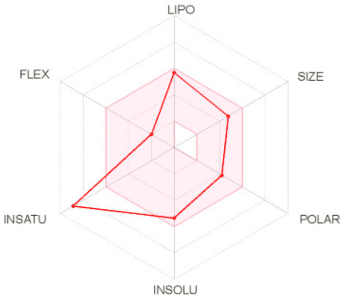

Triclabendazole sulfone</td>
<td>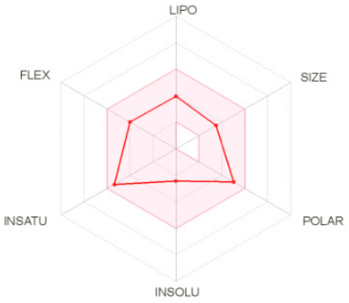

Ricobendazole</td>
</tr>
<tr>
<td>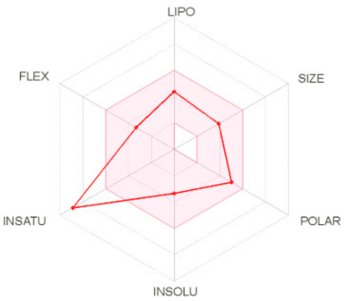

Oxfendazole</td>
<td>Lipophilicity: -0.7 $\leq$ XLOGP3 $\leq$ 5.0
Size: 150 $\leq$ MW (g/mol) $\leq$ 500
Polarity: 150 $\leq$ TPSA (Å^2) $\leq$ 500
Insolubility: 0 $\leq$ Log S (ESOL) $\leq$ 6
Insaturation: 0.25 $\leq$ Fraction Csp3 $\leq$ 1
Flexibility: 150 $\leq$ N° rotatable bonds $\leq$ 500</td>
</tr>
</table>
